# Abiotrophia defectiva Endocarditis Complicated by Stroke and Spinal Osteomyelitis

**DOI:** 10.7759/cureus.56904

**Published:** 2024-03-25

**Authors:** Kevyn Niu, Yizhi Lin

**Affiliations:** 1 Internal Medicine, Hospital Corporation of America (HCA) Florida Blake Hospital, Bradenton, USA

**Keywords:** osteomyelitis, acute cva, endocarditis, antibiotics, abiotrophia

## Abstract

A 67-year-old male with coronary artery disease and aortic stenosis after coronary artery bypass graft (CABG) and aortic valve replacement (AVR) presented after a two-day history of dizziness and frequent falls. Initially, he was found to have a subacute infarct of the left temporal lobe, osteomyelitis of the lumbar spine, and an aortic valve vegetation. Further investigations demonstrated gram-positive bacteremia, and, eventually, the causative organism was identified as *Abiotrophia defectiva*. He was treated with penicillin and gentamicin in the inpatient setting and then discharged with outpatient intravenous (IV) ceftriaxone for the remainder of the four-week antibiotic course. He did not suffer complications after initiating therapy and recovered. We wish to raise awareness of the existence and complications that can result from *A. defectiva* endocarditis and encourage further research into effective antibiotic treatment. *A. defectiva* endocarditis may lead to neurological and orthopedic infective sequelae; understanding and awareness of *Abiotrophia *spp. infections are important to ensure effective treatment of endocarditis.

## Introduction

*Abiotrophia defectiva* is a nutritionally deficient *Streptococcus *species. that has significant potential for endovascular damage, and it is implicated in many cases of culture-negative endocarditis [1]. Because of its fastidious nature, diagnosis and treatment can typically be delayed, which can lead to suboptimal outcomes. *A. defectiva* has been implicated in the distal embolization of organs, leading to multiorgan failure [2]. We present the rare case of *A. defectiva* endocarditis, which led to simultaneous anterior choroidal artery infarction with spinal osteomyelitis and discitis.

## Case presentation

**Figure 1:**
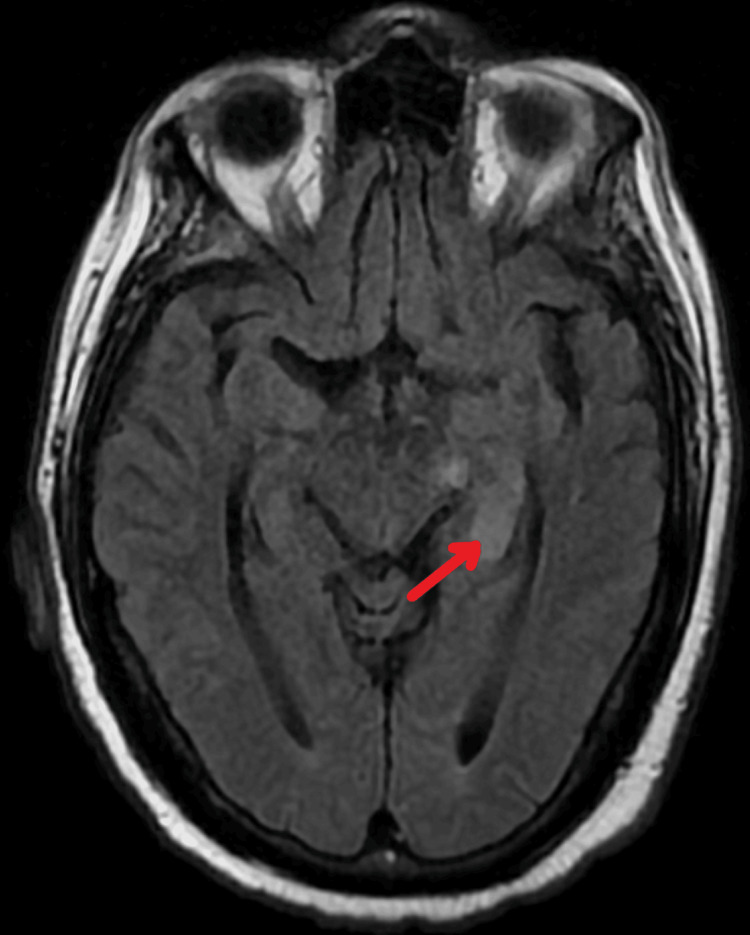
T2-weighted MRI brain. The red arrow indicates the area of subacute infarct in the left temporal lobe of the brain.

We present a case of a 67-year-old male with a past medical history of mitral valve prolapse and aortic stenosis treated with aortic valve replacement (AVR), coronary artery disease treated with coronary artery bypass graft (CABG), degenerative disc disease, and essential hypertension. He did not have any additional risk factors for endocarditis, such as intravenous substance abuse or recent dental procedures. He presented to the hospital with a two-day course of dizziness and frequent falls. He reported multiple falls over three months that had worsened recently. Additionally, he reports incidental right-sided hearing loss, as well as night sweats, anorexia, and a 26-lb weight loss during this three-month period. He denied any head trauma, focal weakness, or bowel/bladder incontinence. No evidence of infective endocarditis was found on physical examination, such as Janeway lesions or finger clubbing. Vital signs were stable on admission. Laboratory studies were positive for elevated leukocytes at 13.6 white blood cells (WBCs) and hyponatremia at 126 mEq/L. A CT imaging of the head demonstrated chronic microvascular disease with no acute intracranial abnormalities. Initial blood cultures were positive for gram-positive cocci in chains, and empiric vancomycin was initiated. An MRI brain demonstrated a subacute infarct in the medial aspect of the left temporal lobe, likely in the anterior choroidal artery (Figure 1).

**Figure 2:**
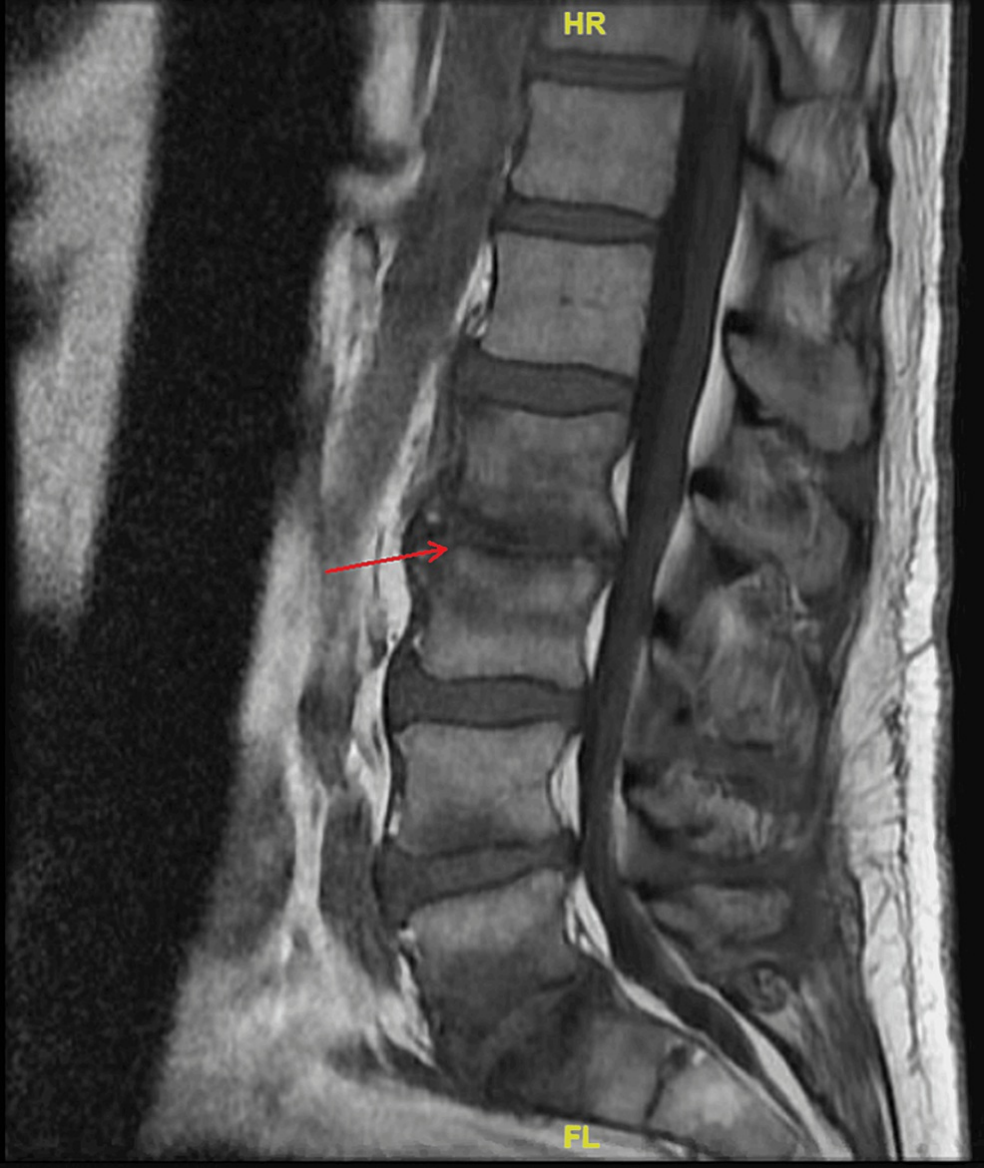
T1 sagittal MRI L-spine. The red arrow at the L4-L5 level demonstrates marked marrow signal changes suggestive of osteomyelitis/discitis.

A CT angiogram of the head and neck revealed a right posterior communicating aneurysm with arterial sclerotic plaques at the right vertebral artery. An MRI of the T-spine and L-spine were positive for signal changes at L2-3, L4-5, and L5-S1, suggestive of osteomyelitis (Figure 2).

**Figure 3:**
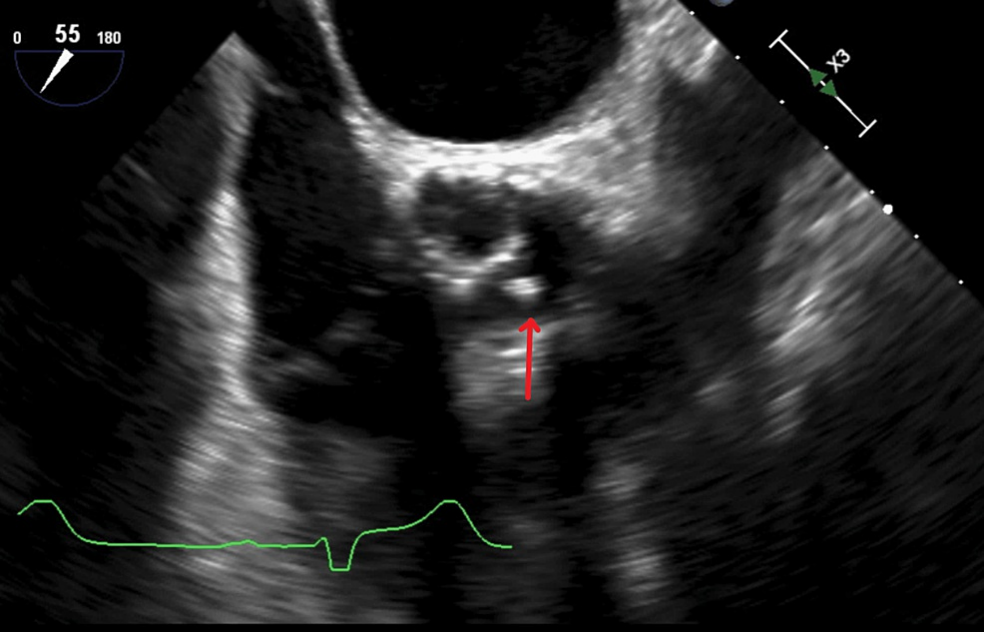
Midesophageal short-axis aortic valve imaging with transesophageal echocardiogram. The red arrow indicates a mobile aortic mass with mild-to-moderate aortic stenosis.

Initial blood cultures returned positive for *A. defectiva*. At this time, vancomycin was replaced with penicillin and gentamicin because of suspicion of left-sided endocarditis with sequelae from septic emboli. A transthoracic echocardiogram (TTE) revealed an ejection fraction (EF) of 55% with severe mitral regurgitation. The TTE revealed an EF of 60-65%, with a probable mobile mass measuring 0.48 cm by 0.35 cm on the aortic valve with undefined origins along with mild-to-moderate aortic stenosis (Figure 3).

Subsequently, *A. defectiva* was confirmed to be the causative organism by two sets of positive blood cultures. Our patient was advised to complete a four-week antibiotic course of penicillin and gentamicin. Sensitivity studies demonstrated that this strain was susceptible to ceftriaxone, and our patient was discharged with an outpatient follow-up.

## Discussion

*A. defectiva* is a nutritionally variant *Streptococcus *(NVS) that is typically found in normal conditions in the oral cavity, GI tract, and genitourinary (GU) system. *A. defectiva* is classified as a gram-positive, nonmotile, facultative aerobe that has the capacity to secrete exopolysaccharides and fibronectin and is thus able to bind to endovascular structures leading to embolic complications. *A. defectiva* requires a complex medium that includes L-cysteine or vitamin B6 [3]; as a result, antimicrobial susceptibility testing remains limited for *A. defectiva*, leading to the difficulty of diagnosis because of the challenges of recovering isolates from specimens [4]. It can be a rare cause of infectious endocarditis and historically has a high incidence rate of valvular complications. The incidence of *A. defectiva* is implicated in approximately 5-6% of streptococcal endocarditis cases, and 1-2% of all causes of infective endocarditis [1], with the most common cause being dental manipulation [5]. Fewer than 150 cases of *A. defectiva *endocarditis have been published in the literature thus far, and, to our knowledge, very few involve simultaneous cerebrovascular accidents with hematogenous seeding to the bone.

Our patient’s medical history was complicated by a previous history of bioprosthetic AVR with CABG, which is the likely nidus for infection. Interestingly, there is low evidence to suggest a predilection for prosthetic valves, in contrast to *Viridans *group streptococci, which tend to favor prosthetic valve infection [5]. Approximately 50% of patients require surgical resection of valvular vegetation [6]. The 2012 EASE trial suggests that patients with severe valvular disease and larger masses would likely benefit from earlier operative intervention and decrease the risk of systemic embolic events [7]. Endocarditis because of *Abiotrophia *spp. typically result in smaller vegetation compared to streptococcal endocarditis [8]. Surgical resection was unnecessary in our patient, who sustained only mild-moderate stenosis of the aortic valve and recovered with antibiotic therapy alone.

Because of the difficulty of treatment, American Heart Association (AHA) guidelines recommend the treatment of *A. defectiva* endocarditis with dual-agent antibiotic therapy similar to the treatment of enterococcal endocarditis. Optimal therapy entails the use of penicillin G with gentamicin, typically for four to six weeks. There is evidence to suggest a synergistic effect when using beta-lactam agents alongside aminoglycosides; however, vancomycin and gentamicin have also been used with varying degrees of success [9]. One retrospective study revealed a 30% success rate with penicillin/gentamicin treatment regimens, with one case requiring vancomycin and ceftriaxone because of the failure of gentamicin therapy [10]. *Abiotrophia *spp. resistance to traditional antibiotic therapy is a field that may require further investigation.

Infections due to *Abiotrophia *spp. are known to cause septic embolization and resultant cerebrovascular ischemic effects at higher rates compared to *Streptococcus *spp. [11]. Previous investigation has demonstrated that early operative (e.g., valvular surgery) interventions are not associated with worsened outcomes in cardioembolic strokes. However, mortality is significantly elevated in patients undergoing operative intervention with hemorrhagic transformation [9]. There have been previously documented cases of *Abiotrophia *spp. endocarditis resulting in hemorrhagic strokes [12]; however, most cerebrovascular complications are cardioembolic and do not involve hemorrhagic transformation. Further investigation into the incidence of hemorrhagic complications resulting from embolic strokes because of *Abiotrophia *spp. is warranted.

In addition to cerebral complications, our patient also sustained osteomyelitis and discitis from *A. defectiva* endocarditis. Puzzolante et al. describe a series of *A. defectiva* osteomyelitis, all of which were treated medically with antibiotic therapy and eventually recovered. Only two cases required spinal surgical intervention [13]. The majority presented with identifiable risk factors for native vertebral osteomyelitis (NVO), including IV drug usage, degenerative spinal disease, and infective endocarditis. Our patient reported a history of chronic degenerative disc disease: this, combined with an obvious nidus of infective endocarditis may have contributed to the development of spinal osteomyelitis. 

The treatment for NVO because of *A. defectiva* is typically four to six weeks of antibiotics: this is identical in nature to our current treatment course. Guidelines per the Infectious Disease Society of America (IDSA) do not specifically point to treatment guidelines for NVO; however, treatment suggestions can be assumed from enterococcal infections. IDSA recommendations for enterococcal infections do not differ significantly from AHA guidelines [14]. Puzzolante et al. suggest that antibiotic treatment length beyond six weeks does not seem to affect the generally favorable clinical outcome in these cases, especially in the context of surgical intervention [13].

## Conclusions

*A. defectiva* is a rare cause of infective endocarditis that is a typical inhabitant of the GI tract. Embolization of an endocarditis nidus is more frequent compared to other infectious species; however, our patient also experienced dissemination to the bone and embolization to the brain, an infrequent occurrence. Our patient recovered without complications after treatment with a course of penicillin and gentamicin in the inpatient setting, and with outpatient management on ceftriaxone. We aim to raise the awareness of clinicians to the existence and complications that can result from A. defectiva endocarditis as well as encourage further research into efficacious antibiotic treatment. We hope this will result in prompt treatment and avoid further complications from *A. defectiva* endocarditis.
